# What the Aftermath of the Global Pandemic Will Mean for Neurologists

**DOI:** 10.3390/neurolint13030030

**Published:** 2021-07-09

**Authors:** Stela Rutovic, Ekaterina Volevach, Hana Maršálková, Ana Isabel Fumagalli, Francesco Corea

**Affiliations:** 1 Department of Neurology, University Hospital Dubrava, Avenija Gojka Suska 6, 10000 Zagreb, Croatia; 2 International Clinical Research Center, St. Anne’s University Hospital, 60200 Brno, Czech Republic; ekaterina.volevach@fnusa.cz (E.V.); hana.marsalkova@fnusa.cz (H.M.); 3 Department of Neurology, Sanatorio Parque, Rosario S2000, Argentina; anisaf7@yahoo.com; 4 Stroke and Neurology Unit, San Giovanni Battista Hospital, 06034 Foligno, Italy; fcorea@gmail.com

**Keywords:** COVID-19, telemedicine, social media

The emergence of the Coronavirus disease (COVID-19) pandemic caused by the SARS-CoV-2 virus has had a widespread public health impact, thus causing a significant socioeconomic burden. Since it was first identified in Wuhan, China, in 2019, it has rapidly spread around the world, with several million reported cases to date.

Consequences on the neurologist’s professional life have been heavy and persistent, starting from new puzzling clinical challenges up to the use of new tools through social media and telemedicine.

Although the respiratory system is the most commonly affected, the SARS-CoV-2 virus can cause various clinical features affecting multiple organ systems. Furthermore, it can invade the central nervous system and cause neurological symptoms. The exact mechanisms of its neurotropic characteristics still need to be investigated, and may include direct infection pathways, immune mediated injury, and brain damage due to systemic hypoxia [1]. In order to provide precise data of the neurological manifestations and outcome of the COVID-19 infection, an international registry (ENERGY) has been created by the European Academy of Neurology (EAN) in collaboration with European national neurological societies and the Neurocritical Care Society and Research Network (NCRN). So far, over 254 sites have registered to the ENERGY study from 69 countries and three continents [2]. This initiative was preceded by a survey of 2343 clinicians on neurological manifestations, completed on 27 April 2020, by the EAN-core COVID-19 task force. It showed that the most common neurological findings included headache, myalgia, anosmia, ageusia, impaired consciousness, psychomotor agitation, daytime sleepiness, encephalopathy, cerebrovascular disease, and dizziness. Less frequent were dysphagia, sleep disorders other than hypersomnia, peripheral nerve damage, seizures, ataxia, meningeal signs, movement disorders, and visual abnormalities [1]. Severe neurological symptoms were more likely to occur in patients with severe COVID-19 disease, or more pre-existing comorbidities such as hypertension, obesity, and diabetes. Long- and short- term post-viral neurological sequelae also need to be monitored as a possibility [3]. It is speculated that SARS-CoV-2 might trigger neurodegenerative diseases like multiple sclerosis (MS), Parkinson’s disease (PD), and narcolepsy. Symptoms of anosmia and ageusia in some patients have been associated with prodromal features of PD. In addition, a significant increase in newly diagnosed mental health disorders like anxiety, depression, insomnia, and dementia has been observed [4].

## 1. The Vaccine Campaigns and Neurological Complications

An array of vaccines against SARS-CoV-2 has been developed with the intention of generating mass immunization, preventing severe disease, and reducing the ongoing health crisis [5].

The availability of COVID-19 vaccines forced neurologists to acquire new skills on the selection of fragile neurological cases undergoing vaccination. Neurological disorders are more frequent in the elderly population with multiple comorbid conditions, or which require treatment with immunomodulatory therapies, which makes neurological patients susceptible to infections with increased risk of severe morbidity and mortality from COVID-19 [3]. The rapid approval of COVID-19 vaccines has caused doubts about their efficacy and safety. Many questions have been raised in patients with multiple sclerosis regarding possible vaccine-induced disease exacerbation and potential interactions between the COVID-19 vaccines and different disease-modifying therapies (DMTs). Studies have not shown clear evidence that COVID-19 vaccines increase the risk of MS activity. A normal SARS-CoV-2 vaccine response is expected in patients treated with IFN-β, teriflunomide, dimethyl fumarate, glatimer acetate, and natalizumab. Attenuated response may be expected in patients taking alemtuzumab, cladribine, mitoxantrone during the depletion phase of treatment, as well as in patients treated with sphingosine 1-phosphate (S1P) modulators, ocrelizumab, rituximab, and patients with lymphopenia during dimethyl fumarate treatment. So far, vaccination with non-live COVID-19 vaccines is recommended for MS patients. Decisions about vaccination should be made carefully and individually for every patient, with special emphasis on selection of optimal timing of vaccination [6].

Studies investigating relations of PD and COVID-19 have shown that patients with PD are not more susceptible to get infected with COVID-19. However, they have elevated risk for experiencing more severe respiratory disease form, especially in advanced PD, as well as increased risk of mortality from COVID-19 compared to general population. Furthermore, both motor and non-motor symptoms of PD can worsen as a result of COVID-19. Vaccines may prevent severe forms of COVID-19, but it has also been questioned whether their use in safe in patients with PD, or if they interfere with PD therapy. Based on current evidence, is seems that vaccination in this group is safe and effective. It is not expected for the vaccines to worsen PD, or interact with therapy, with no different side effects than in general population. Therefore COVID-19 vaccination is recommended for patients with PD, but with special caution for the subgroup of terminally ill elderly persons with PD living in long-term care facilities [7].

Similarly, patients with neuromuscular diseases (NMD) are at increased risk of severe COVID-19 manifestations, especially those with cardiac or respiratory dysfunction. Some of them are on immunosuppressive or immunomodulatory therapies, with increased susceptibility to infections. The major concerns regarding vaccination in this patient group included vaccine-related worsening of the underlying disorder, triggering of new autoimmune NMD, or suboptimal vaccine efficacy. Available evidence of COVID-19 vaccine safety is scarce, but it seems that benefits of vaccination outweigh the possible risks [8].

So far, multiple adverse effects following COVID-19 vaccination have been observed. The most frequent local reaction is mild-to-moderate pain at the injection site. Systemic side effects include fatigue, myalgia, tiredness, and fever. In addition, a few cases of anaphylactic reaction have been reported. Several neurological side effects have also been reported, such as headache, dizziness, paresthesia. Some more serious adverse effects include transverse myelitis, Guillain–Barré syndrome, Bell’s palsy, cerebral venous sinus thrombosis, and ischemic stroke [6,8].

Several videos have been released on social media platforms about new-onset neurological symptoms after COVID-19 vaccination, such as continuous movements of the trunk and limbs or walking difficulties consistent with functional neurological disorder. The popularity of these videos on social media, manifested with millions of views, has been one of factors contributing to vaccine hesitancy [9].

Vaccine hesitancy is especially pronounced in patients with autoimmune diseases who fear that a vaccine might trigger disease exacerbation. On the other hand, older patients and those with more comorbidities are more willing to be vaccinated. It has been shown that patients who are undecided would consider being vaccinated under recommendation of their physician [10]. Understanding patients’ perspectives and adequate communication are crucial, since physicians play a pivotal role as providers of relevant information about the benefits of vaccination.

## 2. The Telemedicine Challenge

The 2020 COVID-19 pandemic boosted, like no other event (e.g., earthquakes, epidemics, war, natural disasters), the implementation of Information Communication and Technology (ICT) resources in health care. Many observed the changes of neurological diseases care pathways undertaken at regional healthcare institutions starting in Italy after the first wave of the COVID-19 pandemic, concluding that telemedicine is one of the more promising answers to approach neurological care in pandemic times [11]. The implementation of the larger Telestroke networks, for example, may reduce futile hospital transfers.

We observed how international medical authorities, in late December 2020 and at the beginning of 2021, released various statements and recommendations to formalize the framework of digital medicine for both device selection and regulatory measures. National and international healthcare authorities depicted their “strategies” in various ways to encourage the deployment of telemedicine. These are long-term plans that will be relevant for neurologists in all settings, from primary care to hospital-based practice and rehabilitation.

The US healthcare authorities showed a longstanding commitment to support regulatory science for digital health, already released in 2017 with the Digital Health Innovation Action Plan [12]. Moreover, in 2020, the activity was strengthened with the preparation of the Digital Health Center of Excellence (DHCoE), a specific office within the FDA [13].

In 2018, European regulatory authorities presented a more timid, even if broad, list of policy priorities for the EU Commission plan for the years 2019–2024. This was built upon previous initiatives enhancing the creation of a Digital Single Market, starting from the assumption that the digital transition should benefit everyone, putting people first and opening new business opportunities [14]. One of the seven flagship initiatives of the Europe 2020 strategy adopted by the Commission is the Digital Agenda, which has the simple diffusion of wideband internet as a core point in many countries. Health is one of the sectors included in this agenda, given the potential benefits that digital services could offer citizens and enterprises in this area [15]. The more advanced European legislation in the area is the Digital Healthcare Act (Digitale-Versorgung-Gesetz or DVG) released in Germany. The Digital Health Application (DIGA) depicts how reimbursement is the key point for digital health solutions, whose importance have been highlighted in the pandemic. The DIGA statement entitles all subjects covered by statutory health insurance to receive reimbursement for certain digital health applications. Devices are described according to their general risk profile and their subtype of technology. After a formal check by the insurer, a prescription by the physician is always needed. The German digital act is today the clearest framework available [16].

The direction given by political authorities seems clear: it is now up to the neurologist understanding as to how to take part in the transition.

## 3. The Pandemic and Social Media

As early as 2017, the World Health Organization (WHO) Regional Office for Europe highlighted the importance of using e-health (and social media) in promoting public health [17]. Despite this, there is still no single strategy for health promotion through social media, analyzing its effectiveness; public health professionals remain incompetent in the use of digital technology. Therefore, The National Commission for Health Education, Inc. (NCHEC) and the US Society for Public Health Education (SOPHE) released guidelines for effective use of social media. These guidelines include recommendations to actively engage influential and interested parties to expand advocacy on social media, identify the most effective social media, and develop different types of social media activities and methods for assessing their effectiveness [17].

However, examples from practices show that mentioned best practice is often not followed. For example, despite large financial investments by the Centers for Disease Control (CDC) in public health in 2016, funding for the development of social media was poor [18]. Moreover, the Brazilian Ministry of Health was unable to achieve public engagement due to posting repeating text context and institutional content [19]. Notwithstanding, using a professional marketing approach, government accounts can be promising for promoting health. An Australian empirical study of the Twitter accounts of non-profit, for-profit and governmental organizations showed that the largest number of retweets (70%) of health advances in 2012 were from government organizations [20]. The Portuguese government organization has also managed to maintain public interest in public health topics in their Instagram account [19]. Common characteristics of retweeted and shared messages were as follows: availability of practical health information that can be applied immediately, temporary relevance of information, urgent need to share information with others, individual language style using words such as “our”, “your”, inclusion of rhetorical questions [20].

Despite the mentioned opportunities of official organizations to become popular among the general public, stroke organizations primarily use social media for coverage institutional and academic news. For example, an analysis of the Twitter hashtag #ESOSS17 showed that “118 tweets were sent generating >50,300 impressions”. In addition, the hashtag @StrokeAHA_ASA (with links to journal articles) has about 6500 followers on Twitter [21]. Stroke awareness activities on their pages take place mostly during the “month of stroke” [21,22]. Despite such a limited use, existing studies show that the promotion of stoke awareness through social media has much more audience coverage than traditional methods, such as lectures, leaflets, etc. For example, the results of Hundt and Chen’s study show that only 192 of participants were interested in measuring their stroke risk during a community-setting awareness program compared to 6010 of participants interested during social media advertising [22]. A study at Mansoura University Hospital (USA) stressed the usefulness of social media for increasing stroke awareness by showing that, out of 830 stroke patients who use social media, 243 patients were aware of their stroke symptoms from social media [23]. These results suggest that the public has an interest in stroke topics on social media and the accounts of influential organizations should be used for this purpose.

It seems that the COVID-19 pandemic has changed the attitude of both organizations and users toward the use of information on social media, increasing their impact on public health. The number of Instagram followers of WHO, WHO Europe, and the Pan American Health Organization increased from 2.6 million followers before COVID-19 to 5.1 million followers one month later (from March to April) [19]. The reason was that the pandemic has made its own adjustments in the field of health care and patient care. For example, the opportunity to see a doctor has sharply decreased, the need for social distancing has increased, and the possibility of social communication with people (for example, friends or relatives) who can give health advice has decreased. At the same time, trust in traditional media (such as radio, television, print, and billboards) controlled by the authorities has declined due to inconsistent information [23].

Because of these changes, social media has become a driving way of sharing, producing, and disseminating information related to health and medicine [8]. However, precisely because of its structure, which allows for the rapid dissemination of information, social media began to spread myths and misinformation about the coronavirus. The necessity to fight against fake information prompted politicians and medical institutions to transfer most of their activity to the social media space [24]. Government agencies began to cooperate with Twitter, Facebook, Instagram, and others to convey health information on behalf of social media [9]. One of the innovative methods that appeared only during the pandemic period was also the ability to disseminate health information using automatic messages in WhatsApp (and other chats) [23].

The COVID-19 pandemic has also changed the way physicians engage in health promotion. Previously doctors were not recommended to speak publicly in social media [25]. The impetus for the change was the spread of false information about the coronavirus: some doctors considered it their duty to refute myths and pass on verified information to the public. Therefore, while formal institutions (CDC, WHO) were underrepresented on social media [18], many physicians who recorded informational videos about the coronavirus had an audience of up to 6 million. Their conversations differed from the usual interviews of physicians in traditional media: physicians-influencers spoke not only about clinical facts, but also used methods of crisis communication, interacted with the public, and eliminated social panic [25].

The increased creditability of social media during the COVID-19 pandemic can be effectively used to start social media stroke awareness campaigns on the national level [26]. In connection with the pre-COVID using of social media in stroke issues (which included more institutional news and advocacy for scientific articles), the question of improving social health marketing in stroke is urgent. For this, it is necessary to increase the competence of stroke professionals in digital technologies and social marketing through, for example, master direct communication with the public, the publication of practical advice, etc. Mentioned activities should consist of the temporary relevance of information and the urgent need to share this information with others. For example, during the COVID-19 pandemic, stroke organizations could interest the public with news about the influence of COVID-19 on stroke prevalence [27]. Such news should be presented in a form that will interest the general public. It should be written in simple language, contain a call for communication or action (for example, rhetorical questions), and include videos or pictures. This way of communicating information can also be used after a pandemic, by linking information about stroke with the problems that people are interested in in everyday life.

The next important “COVID-19 innovation” is the increased involvement of government agencies in health and social media issues. These organizations’ pages on social media are now followed by many people who trust them. Neurology-related institutions could offer them collaborations that can be also beneficial both for government (e.g., maintaining the confidence of the electorate) and for institutions (e.g., highlighting the importance of neurological issues at the national level). Also, direct collaboration with social media on behalf of the state in the issues of stroke awareness (e.g., in America, Facebook has created an application for monitoring health, including blood pressure) can be offered. It is also possible to co-operate with social chats in the field of sending automatic messages (for example, about the FAST method for stroke symptoms recognition). Collaboration with physicians-influencers is also needed. They have a large public reach, promote only verified information, and have a lot of public confidence. During COVID-19, their thematic videos only covered the pandemic. However, after its end, they will probably start to engage the public in other health topics, so as not to lose their audience. They may be invited to collaborate in promoting information on neurological diseases’ awareness. On the other hand, this strategy is only suitable for countries that have physicians-influencers. In other cases, it is possible to collaborate with celebrities who covered health issues on social media during the COVID-19 pandemic.

On the horizon, we see neurology tilting toward a more efficient organization in which digital health is playing a central role. We see the need for neurologists to upgrade their local and global networks (e.g., web-based databases, telemedicine) to face the present and future COVID-19 challenges, as well as maintain capacity on traditional clinical lines.

## Data Availability

Not applicable.
